# Circulating Tumor Cells from Prostate Cancer Patients Interact with E-Selectin under Physiologic Blood Flow

**DOI:** 10.1371/journal.pone.0085143

**Published:** 2013-12-27

**Authors:** Gunjan Gakhar, Vicente N. Navarro, Madelyn Jurish, Guang Yu. Lee, Scott T. Tagawa, Naveed H. Akhtar, Marco Seandel, Yue Geng, He Liu, Neil H. Bander, Paraskevi Giannakakou, Paul J. Christos, Michael R. King, David M. Nanus

**Affiliations:** 1 Deparment of Medicine, Division of Hematology and Medical Oncology, Weill Cornell Medical College, New York, New York, United States of America; 2 Department of Urology, Weill Cornell Medical College, New York, New York, United States of America; 3 Weill Cornell Cancer Center, Weill Cornell Medical College, New York, New York, United States of America; 4 Department of Surgery, Weill Cornell Medical College, New York, New York, United States of America; 5 Department of Biomedical Engineering, Cornell University, Ithaca, New York, United States of America; 6 Division of Biostatistics and Epidemiology, Department of Public Health, Weill Cornell Medical College, New York, New York, United States of America; University of Oslo, Norway

## Abstract

Hematogenous metastasis accounts for the majority of cancer-related deaths, yet the mechanism remains unclear. Circulating tumor cells (CTCs) in blood may employ different pathways to cross blood endothelial barrier and establish a metastatic niche. Several studies provide evidence that prostate cancer (PCa) cell tethering and rolling on microvascular endothelium via E-selectin/E-selectin ligand interactions under shear flow theoretically promote extravasation and contribute to the development of metastases. However, it is unknown if CTCs from PCa patients interact with E-selectin expressed on endothelium, initiating a route for tumor metastases. Here we report that CTCs derived from PCa patients showed interactions with E-selectin and E-selectin expressing endothelial cells. To examine E-selectin-mediated interactions of PCa cell lines and CTCs derived from metastatic PCa patients, we used fluorescently-labeled anti-prostate specific membrane antigen (PSMA) monoclonal antibody J591-488 which is internalized following cell-surface binding. We employed a microscale flow device consisting of E-selectin-coated microtubes and human umbilical vein endothelial cells (HUVECs) on parallel-plate flow chamber simulating vascular endothelium. We observed that J591-488 did not significantly alter the rolling behavior in PCa cells at shear stresses below 3 dyn/cm^2^. CTCs obtained from 31 PCa patient samples showed that CTCs tether and stably interact with E-selectin and E-selectin expressing HUVECs at physiological shear stress. Interestingly, samples collected during disease progression demonstrated significantly more CTC/E-selectin interactions than samples during times of therapeutic response (*p*=0.016). Analysis of the expression of sialyl Lewis X (sLe^x^) in patient samples showed that a small subset comprising 1.9-18.8% of CTCs possess high sLe^x^ expression. Furthermore, E-selectin-mediated interactions between prostate CTCs and HUVECs were diminished in the presence of anti-E-selectin neutralizing antibody. CTC-Endothelial interactions provide a novel insight into potential adhesive mechanisms of prostate CTCs as a means to initiate metastasis.

## Introduction

The development of metastases is hypothesized to initiate via similar mechanisms as used by leukocytes for adhesion and transmigration through blood endothelium [1]. The leukocyte recruitment cascade to endothelium during inflammation involves sequential steps including tethering, rolling, adhesion, and finally transmigration. The initial step of tethering and rolling occurs through dynamic and transient interactions between selectins expressed by endothelial cells (ECs) and their respective ligands present on leukocytes during blood flow causing continuous breaking and formation of receptor-ligand bonds. This cycle leads to the characteristic rolling behavior of leukocytes [2]. In humans, E-selectin has been shown to be primarily responsible for selectin-mediated rolling and tethering [3].

Numerous studies using tumor cell lines and mouse models suggest that endothelial (E)-selectin is also involved in tumor cell adhesion, migration and the development of metastases [4,5]. *In vivo* mouse models have shown that E-selectin promotes metastases [6] and redirects metastases to the liver [7]. Cimetidine, which inhibits the induction of E-selectin expression, significantly decreased liver metastasis of HT-29 colon cancer cells in an athymic mouse model without affecting the primary tumor [8]. Furthermore, E- and P-selectin deficient mice injected with HT-29 tumor cells showed a significant decrease in the number of lung metastasis compared with wild type mice [9].

The link between E-selectin and metastasis led to many studies characterizing selectin ligands on tumor cell surfaces. Selectin ligands are specific glycoproteins, which become functional after post-translational modification by glycosyltransferases and sulfotransferases. The presence of sialyl-Lewis X (sLe^x^) and sialyl-Lewis a (sLe^a^), carbohydrate epitopes of selectin ligands [10,11] is frequently associated with cancer progression and poor prognosis [12,13]. Studies also suggest that the tropism of PCa cells to bone is attributed to the interactions between E-selectin expressed on bone marrow endothelial cells (ECs) and E-selectin ligands present on PCa cells [14]. 

The evidence implicating the role of E-selectin and its ligands in tumor metastasis are derived from studies using tumor cell lines but have never been confirmed in circulating tumor cells (CTCs) derived from patients. We report here using *ex vivo* conditions that CTCs isolated from men with castration-resistant prostate cancer (CRPC) demonstrate physical interactions, predominantly tethering and firm adhesion, with E-selectin-coated surfaces and E-selectin expressing ECs during physiological blood flow. Additionally, CTC/E-selectin interactions showed a significant correlation with the clinical response of the patients to therapy. These interactions were diminished in the presence of anti-E-selectin neutralizing antibody. Furthermore, we found variable expression of sLe^x^ on CTCs, suggesting that likely not all CTCs contribute to metastases. 

## Materials and Methods

### Cell lines

PC3, C4-2, LNCaP, MDA PCa 2b (MDA), and KGI cells are from ATCC (Manassas, VA, USA). PCa cell lines PC3, C4-2, and LNCaP were maintained in RPMI supplemented with 10% FBS, and MDA PCa 2b (MDA) was maintained in F-12K media supplemented with 20% fetal bovine serum (FBS), 10 ng/ml EGF, 0.005mg/ml insulin, 100 pg/ml hydrocortisone, 25 ng/ml cholera toxin, 45 nM selenious acid, and 0.005 mM phosphoethanolamine. KG1 cells (acute myelogenous leukemic cell line) were cultured in IMDM media supplemented with 20% FBS.

### Ethics Statement and Patient sample collection

Under a Weill Cornell Medical College Institutional Review Board (IRB) approved protocol, 31 peripheral blood samples were obtained from patients with CRPC and 10 peripheral blood samples were taken from healthy donors following written informed consent. Blood was obtained in either Ficoll-paque tubes (7.5 ml blood each) or BD Vacutainer tubes (2.7 ml blood each; Becton-Dickinson) containing 2.3% sodium citrate anticoagulant. De-identified clinical information was obtained. Determination of tumor state (clinical progression or clinical response) was determined by 2 independent clinicians using standard criteria.

### Surface Labeling with anti-PSMA monoclonal antibody J591

MDA cells were trypsinized, centrifuged, and incubated in Hank’s balanced salt solution (HBSS)/10mM HEPES/2mM CaCl_2_/0.5% human serum albumin (buffer I). Monoclonal antibody J591 [15] that recognizes the external domain of prostate specific membrane antigen (PSMA) conjugated with Alexa fluor-488 (J591-488) was added to MDA and PC3 cells (alone and cells spiked in normal healthy blood) at 20 µg/ml for 30 min at room temperature on a rotator, centrifuged at 1500 rpm for 5 min, and the pellet resuspended in RPMI media. Peripheral blood mononuclear cells (PBMCs) isolated from normal healthy blood by ficoll density-based centrifugation were similarly processed, and placed onto BD cell-tak (BD Biosciences, San Jose, CA) coated coverslips by cytospin. PBMCs were fixed using 2% formaldehyde (Tousimis, Rockville, MD) and stained with mouse anti-CD45 antibody (1:200, Clone 2D1, BD Pharmingen, San Jose, CA), washed with PBS and incubated with goat anti-mouse Alexa fluor (AF)-594 secondary antibody, washed and counterstained with DAPI.

### Enrichment of CTCs by anti-CD45 immunomagnetic bead depletion

To enrich for CTCs, PBMCs isolated from peripheral blood by Ficoll density-based centrifugation were subjected to negative selection for leukocytes using anti-CD45 immunomagnetic beads (Life Technologies, Grand Island, NY). PBMCs were washed twice with 2% RPMI/4mM MgCl_2_/1mM CaCl_2_ buffer (buffer II) and incubated with pre-washed anti-CD45 immunomagnetic beads in 1 ml buffer I for 20 min on a rotator. After incubation, 35 ml buffer I was added and cells were kept on a magnet for 10 min at 4°C. Cells were centrifuged at 400 g for 12 min and anti-PSMA J591-488 antibody was added for 40 min, centrifuged and resuspended in buffer I and rolling assays were performed. For immunostaining experiments, following anti-CD45 immunomagnetic depletion, cells were resuspended in 500 µl of 2% RPMI medium and cytospun onto positively charged glass slides (Thermo Scientific, Asheville, NC). Slides were stored at -20°C for subsequent immunostaining.

### Flow-Based Microtube Assay

Briefly, sterile 50 cm length of 300 μm ‘D’ microrenathane tubes (Braintree Scientific, MA) were incubated with 10 mg/mL protein-G solution for 1.5 h, followed by a 2 h incubation with 10 µg/mL human recombinant IgG E-selectin [16] (R&D Systems, Minneapolis, MN) and blocked with 5% milk for 1 h. All incubations were performed at room temperature. Control tubes were either not incubated with E-selectin or incubated with 10 µg/ml human recombinant IgG L-selectin (R&D Systems, Minneapolis, MN). Coated microtubes were mounted on an inverted microscope equipped with a Zeiss AxioCam MRm camera. A syringe pump (KDS 230, IITC Life Science, Woodland Hills, CA) was used to control the shear stress of the cell suspension. A known concentration of 1 x 10^6^ unlabeled or J591-488 labeled MDA cells diluted in buffer I was perfused over the surface at a shear stress of 0.5 dyne/cm^2^ for 3 min following which cells were perfused at shear stresses ranging from 0.5-8 dyn/cm^2^. Videos were recorded for 30 sec at 10 random locations along the length of each microtube. 

For measurement of patient CTC interactions with E-selectin, PBMCs obtained from 21 CRPC patient samples after anti-CD45 depletion and six patient samples without anti-CD45 depletion were labeled with J591-488 and flowed over E-selectin-coated microtubes at 0.6 dyn/cm^2^. J591-488 labeled CTCs were visualized using 488 nm laser wavelength. A series of continuous 45 sec videos were recorded using Axiovision time-lapse module (Carl Zeiss, Thornwood, NY). All tubes and pipette tips were pre-coated with 0.5% human serum albumin to block non-specific binding of CTCs. 

### Laminar Flow analysis for CTC-Endothelial cell interactions

For CTC-endothelial interactions, a parallel-plate flow chamber was used [17]. HUVECs (a gift of Dr. Shahin Rafii) were cultured in M199 media, 1 M Hepes, 20% FBS, 5 mg/ml heparin, 100 µg/ml endothelial cell growth factor, and L-glutamine. E4ORF1-transduced HUVECs were prepared and maintained as previously described [18]. MDA cells or CTCs derived from four CRPC patients were perfused over confluent monolayers of human umbilical vein endothelial cells (HUVECs) grown in a 35 x 10 mm tissue culture dishes (Corning Inc., Corning, NY). HUVECs were first stimulated with 50 ng/ml IL-1β (Peprotech, Rocky Hill, NJ) for 4 h. IL-1β-stimulated HUVECs treated with 30 µg/ml neutralizing anti-E-selectin (68-5H11, BD Pharmingen, CA) for 1 h at 37°C incubator, unstimulated HUVECs, and human anti-mouse ICAM-1 antibody (Clone BBIG-I1, # BBA3 R and D systems) were used as controls. MDA cells or CTCs were resuspended in buffer I and perfused into the parallel-plate flow chamber over HUVECs. MDA cell rolling was assessed at different shear stresses ranging from 0.5-4 dyn/cm^2^ performing three independent experiments. For rolling measurements in CTCs derived from PCa patients, cells enriched by anti-CD45 immunomagnetic depletion and labeled with anti-PSMA J591-488, were perfused over HUVECs at 0.6 dyn/cm^2^ shear stress. As indicated in the results, a subset of rolling assay experiments were also performed using Bioflux Microfluidics technologies (Fluxion Biosciences, San Francisco, CA).

### Offline analysis of E-selectin-mediated interactions

“Rolling” cells were defined as any cell translating along the tube surface for longer than two seconds at a velocity less than 50% of the hydrodynamic free stream velocity of a non-interacting cell near the tube wall [19]. Rolling velocity was quantified by recording the amount of time (t) required for a rolling cell to move across a known distance (d). Rolling velocity v= d/t. 

To assess interactions of patient-derived CTCs with E-selectin, CTCs were categorized into three classes: 1) Stable adhesion CTCs (i.e., stuck to the surface for more than five sec); 2) Rolling/Tethering CTCs (i.e., tethering refers to CTCs which attach to the surface, then detach in less than five sec and reattach again); and 3) Non-adherent CTCs (CTCs which did not roll/tether or adhere to the surface).

### Immunofluorescent staining of cell surface markers

Following enrichment of PBMCs from PCa patients, isolated cells were attached to the glass slides by cytospin, fixed in 2% formaldehyde (Tousimis, Rockville, MD) for 20 min, and washed 3X with PBS. Cells were blocked with 2% BSA for 1 h, and incubated with primary antibodies rat anti-sLe^x^ (1:10, HECA-452, BD Pharmingen, San Jose, CA) or rabbit polyclonal anti-CXCR4 (1:100, Novus Biologicals, Littleton, CO) mAbs for 1 h, and incubated with secondary Abs (goat anti-rat AF594 and goat anti-rabbit Dylight 405) for 1 h, incubated in AF-488 and AF-647 conjugated primary antibodies for anti-PSMA (humanized) and anti-EpCAM (mouse, Clone 9C4, BD Biosciences), respectively and mounted on a coverslide with freshly prepared mowiol (Calbiochem, Billerica, MA). All incubations were performed at room temperature followed by 3X wash with PBS plus 0.5% BSA. For immunofluorescent staining, MDA, PC3, KG1cells, and PBMCs obtained from normal healthy donors were used as negative and positive controls for different proteins. Control cells were seeded on BD Cell-Tak coated coverslips and processed similarly as patient samples. Quantification of sLe^x^ expression data was obtained using Metamorph^TM^ software (MDS Analytical Technologies, Sunnyvale, CA) and measuring the mean fluorescence intensity (MFI) per pixel [20]. The background intensity, determined by selecting an area lacking CTCs, was subtracted from these values for each image. For these measurements, Z-stacks of the acquired images were used taken by LSM 700 Zeiss Observer.Z1 (Carl Zeiss, Microimaging Inc, Thornwood, NY).

### Western blot and immunofluorescene for E-selectin expression in HUVECs

Both 1° and E4ORF1 HUVECs were stimulated with IL-1β for 4h. After IL-1β stimulation, both unstimulated and stimulated cells were washed three times with ice-cold PBS. Cells were resuspended in lysis buffer (150 mM NaCl, 10 mM Tris HCL, pH=7.5, 5 mM EDTA) containing Protease Inhibitor Cocktail tablet (1 tablet/10 ml lysis buffer; Roche). Protein concentrations of cell lysate were determined by Bradford method. Cell lysate was diluted in reducing 6X sample buffer (12% w/v SDS, 0.06% w/v bromophenol blue, 47% v/v glycerol, 0.5 M Tris, pH=6.8, bring the volume to 10 ml with distilled water) and separated on 10% SDS-PAGE gel. Resolved proteins were transferred to polyvinylidene difluoride membrane (Bio-Rad, Inc., Hercules, CA) and blocked in 5% milk for 1 h at room temperature. Membrane was cut and then incubated with mouse anti-E-selectin (1:500, 68-5H11, BD Pharmingen, San Jose, CA) and mouse anti-β-Actin (1:10,000, Sigma Aldrich) for overnight at 4°C. Immunoreactivity was visualized by the LI-COR Odyssey Infrared Imaging System (LI-COR Biosciences). For immunofluorescence, both 1° and E4ORF1 HUVECs were stimulated with IL-1β for 4h and both unstimulated and IL-1β-stimulated HUVECs were fixed, permeabilized with 0.1% Triton-X 100, and immunostained with mouse anti-E-selectin monoclonal antibody (2.5 µg/ml, 68-5H11, BD Pharmingen, San Jose, CA) overnight at 4°C. HUVECs were stained with Alexa fluor goat anti-mouse 488 nm antibody (1:500, Molecular Probes) for 1 h at room temperature. HUVECs were washed and counterstained with DAPI.

### Statistical Analysis

Descriptive statistics are presented with histograms showing mean + standard deviation (SD). Means were compared by the two sample t-test with appropriate variance calculator. Box plots were made and the non-parametric Wilcoxon rank-sum test was performed to compare the distributions (i.e., median, range) between groups. A minimum of three independent experiments were performed with cancer cell lines. The Fisher’s exact test was performed to assess the relationship between patient CTC/E-selectin interaction status and clinical progression status. A p-value of *<0.05* was considered statistically significant in all of the analyses. All analyses were performed in STATA Version 10.0 (StataCorp, College Station, TX).

## Results

### Labeling of prostate tumor cells with anti-PSMA J591-488 antibody

**Figure 1:**
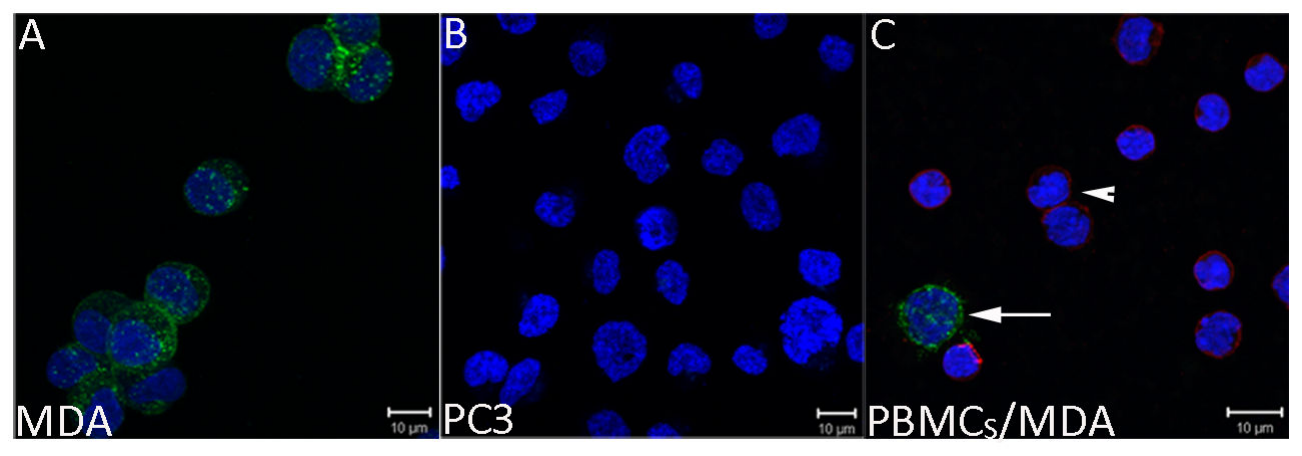
Specificity of PSMA expression on prostate cancer cells by confocal microscopy. Cancer cells were plated either alone (A, B) or in the presence of healthy donor derived PBMCs (C). Cells were labeled with J591-488 antibody (green) at room temperature, then fixed and immunostained for CD45 (red) and DAPI (blue). **A**) MDA cells are positive for PSMA expression. **B**) PC3 cells are negative and therefore, lack the PSMA expression. **C**) Right subfigure shows the specific expression of PSMA by MDA cells while blood cells express CD45. PBMCs isolated from normal healthy donors were mixed with MDA cells and processed as in A, B. PSMA-positive MDA cancer cells are clearly and specifically depicted (white arrow) among CD45-expressing leucocytes (arrowheads) in the mix. *Green= PSMA, Red= CD45, and Blue=DAPI*.

To study CTCs isolated from patients with CRPC, we took advantage of the fact that >90% of PCa cells express PSMA on their cell surface [21,22], and used mAbJ591-488, which recognizes an external domain of PSMA and is rapidly internalized following binding to cell-surface PSMA, to identify PCa CTCs [15,23].. MDA cells expressing PSMA and PC-3 cells (PSMA negative control) were added separately to normal healthy blood, and the mixture was incubated with mAbJ591-488. After incubation, the PBMC layer containing cancer cells were collected. As expected (Figure 1A), MDA cells expressing PSMA internalized mAbJ591-488 and were easily visible under fluorescent microscopy, while mAbJ591-488 did not bind to PC3 cells (Figure 1B). Blood spiking experiments with MDA and PC3 were carried out six times using different healthy donor blood. No PSMA expression was detected in surrounding leukocytes that were identified by immunostaining with anti-CD45 antibody (Figure 1C). PSMA expression was also not observed in PC3 cells mixed with blood (data not shown). These results suggest that mAbJ591-488 can specifically label PCa cells and can thus be used to label PCa CTCs.

### E-selectin-dependent adhesion of unlabeled and anti-PSMA J591-488 labeled prostate tumor cells

During an inflammatory response, leukocytes adhere to the vascular endothelium by interactions between the adhesion molecules present on the luminal side of the vascular endothelium and complementary ligands present on leukocytes. These interactions result in the transient, dynamic adhesion (rolling) of cells to the vascular wall [2]. Rolling is observed due to continuous breaking and formation of receptor-ligand bonds at the rear end of the cell and the front edge of the cell, respectively. To investigate whether E-selectin-dependent interactions occur in prostate CTCs, we first used MDA, PC3, LNCaP, and C4-2 PCa cell lines to assess their rolling behavior in the presence of E-selectin-coated microtube surfaces. Out of the four PCa cell lines studied, we only detected robust rolling behavior in MDA cells (data not shown), consistent with prior studies [24,25]. 

**Figure 2:**
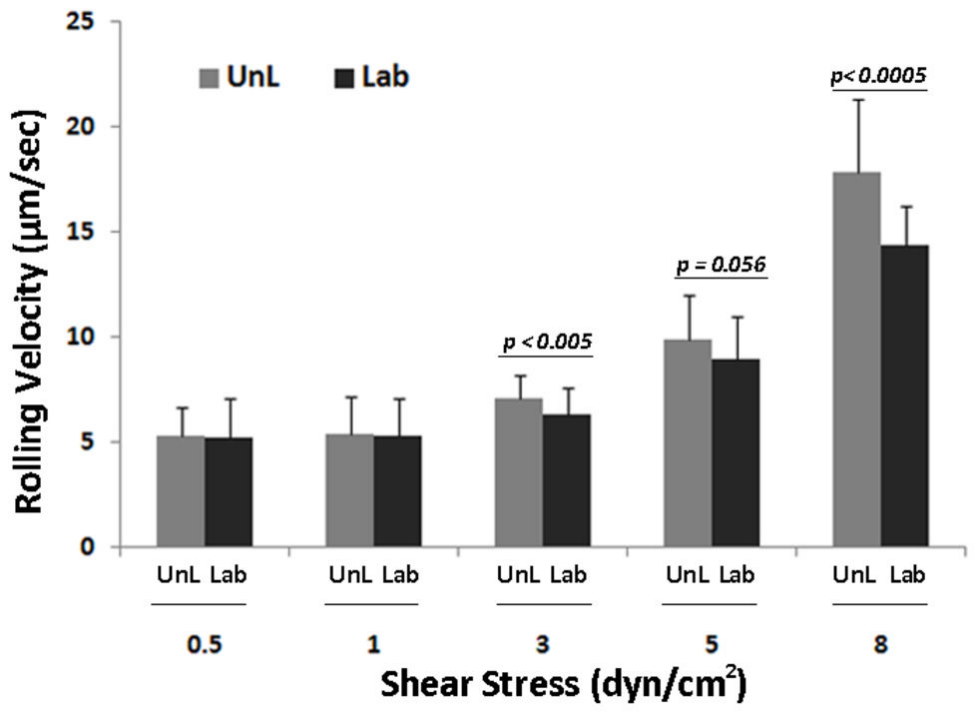
Rolling velocity of unlabeled and anti-PSMA labeled MDA cells at different shear stress. MDA cells were labeled with mAb J591-488 for PSMA. After labeling, 10^6^ J591-488 labeled MDA cells were perfused at 0.5, 1, 3, 5, and 8 dyne/cm^2^ shear stress. Similarly unlabeled MDA cells were also perfused through E-selectin coated microtubes. Ten videos were taken at different lengths of the microtube for each shear stress. Rolling velocity was measured for both unlabeled and anti-PSMA J591-488 labeled MDA cells. Figure shows no significant difference in the rolling velocity between unlabeled and anti-PSMA J591-488 labeled MDA cells at shear stress ranging from 0.5-1 dyn/cm^2^. The mean rolling velocities at 0.5 dyn/cm^2^ were 5.27+ 1.38 and 5.23 + 1.85 µm/sec in unlabeled and J591-488 labeled MDA cells, respectively. At higher shear stresses, a significant difference was observed between the two categories of MDA cells. Histogram shows the results from three separate experiments combined together. UnL= unlabeled MDA cells, Lab= J591-488 labeled MDA cells. Data is represented as Mean + SD.

Monoclonal antibody J591 is internalized following cell-surface binding suggesting that PCa CTCs can be labeled *ex vivo* and studied for CTC-endothelial interactions. However, to rule out the possibility that J591-488 binding and internalization alters rolling behavior, we labeled MDA cells with J591-488 and compared the rolling behavior with unlabeled MDA cells at shear stresses ranging from 0.5-8 dyn/cm^2^. The mean rolling velocity of unlabeled and J591-488-labeled MDA cells at 0.5 dyn/cm^2^ was 5.27 + 1.28 μm/sec and 5.23 + 1.85 μm/sec (Figure 2, p=0.88, NS). We did observe a small but statistically significant difference in the mean rolling velocities of unlabeled versus J591-488 labeled MDA cells at 3 (7.04 + 1.15 vs 6.33 + 1.22 µm/sec, *p<0.005*) and 8 (17.81 + 3.51 vs 14.38 + 1.83 µm/sec, *p<0.0005*) dyn/cm^2^ shear stress. Therefore, based on our results and previous studies, we conducted experiments at 0.6 dyn/cm^2^, a widely applied physiologic shear stress to measure rolling behavior [17]. Also, it has been shown that tumor cells roll and tether at lower shear stresses (

< 3 dyn/cm^2^) [26]. These data suggest that CTCs can be labeled with J591-488 and that fluorescent labeling of CTCs does not affect rolling behavior at lower shear stresses. Of note, we did not detect any rolling behavior either in the absence of E-selectin at any shear stress examined or in the presence of human recombinant L-selectin protein (data not shown), suggesting that E-selectin is required for the rolling behavior. Videos S1 and S2 show the rolling behavior of unlabeled and J591-488-labeled MDA cells

. 

### E-selectin-mediated interactions in CTCs derived from prostate cancer patients

We next determined whether CTCs derived from PCa patients would interact with E-selectin similar to MDA PCa cells. PBMCs from ~6 ml (5.4-7.5) of whole blood were obtained from 17 individual patients (27 blood samples) with metastatic PCa, and incubated with mAb J591-488. Following labeling, 21 samples underwent anti-CD45 depletion; while in 6 samples, no anti-CD45 depletion was performed. We first ran six patient samples without anti-CD45 depletion. Subsequently, to avoid contaminating leukocytes that could potentially decrease the chance of CTCs interacting with the surface, we employed anti-CD45 depletion to enrich for the CTC population. The recovery rate of this technique was 60-70% using PSMA expressing PCa cells (data not shown). Each patient sample was perfused over E-selectin-coated microtube surfaces at 0.6 dyn/cm^2^ generating a wall shear rate of 60^-s^, which is within the range of physiological wall shear rate in postcapillary venules [27]. PCa CTCs labeled with mAb J591-488 were identified in 23 samples, and the median number of CTCs detected using offline video analysis was nine (range 0-270) (Table 1). Nine of 23 (39.1%) and 10/23 (43.5%) of patient samples showed rolling/tethering and stable adhesion, respectively. The E-selectin mediated interactions observed in prostate CTCs comprised mostly tethering and stable adhesion with very few CTCs showing rolling behavior. When we assessed clinical state at the time of sample collection and processing, we noted that CTCs from samples of patients experiencing a clinical response were significantly less likely to have E-selectin-mediated interactions compared to CTCs from clinically progressing patients (Table 1; 0.0% vs. 61.9%, respectively, *p*=0.016). Anti-CD45 depletion did not have an effect on E-selectin-dependent interactions in CTCs (Table 1, sample 1, 13, 17, 21, 22, 25); although, we cannot completely rule out the possibility that some CTCs were lost during anti-CD45 depletion process. Video S3 shows a video from one patient sample demonstrating the interaction of CTCs with E-selectin. These results provide direct physical evidence that prostate CTCs expressing PSMA interact with E-selectin and suggest that prostate CTCs possess E-selectin ligand(s) (ESLs) on their cell surfaces. 

**Table 1:** Interactions between CTCs derived from metastatic PCa patients and E-selectin-coated microtube surfaces.

**Patient No.**	**Sample No.**	**Rolling and Tethering**	**Stable Adhesion**	**Total**	**Clinical History at time of sample collection**	**Clinical Response at time of sample collection**
1	*1	3	3	29	Bone and LN metastases following progression on docetaxel and investigational therapy currently on ketoconazole/hydrocortisone	Progression
1	2	1	3	10	Bone and LN metastases following progression on docetaxel and investigational therapy currently on ketoconazole/hydrocortisone	Progression
1	3	28	14	270	Bone and LN metastases following progression on hormonal therapy, docetaxel, and investigational therapy currently on abiraterone/prednisone	Progression
1	4	0	0	72	Bone and LN metastases following progression on docetaxel and abiraterone currently on carboplatin/paclitaxel	Responding
1	5	0	0	12	Bone and LN metastases following progression on docetaxel and abiraterone currently on carboplatin/paclitaxel	Responding
2	6	0	0	9	Bone and LN metastases treated previously with hormonal therapy and docetaxel on investigational therapy	Progression
2	7	0	0	4	Bone and LN metastases treated previously with hormonal therapy and docetaxel on investigational therapy	Progression
3	8	0	3	5	Bone metastases following multiple lines of hormonal therapy	Progression
3	9	0	0	9	Bone metastases following multiple lines of hormonal therapy on docetaxel	Responding
4	10	0	1	2	Bone, LN, liver and lung metastases previously treated with docetaxel and abiraterone currently on carboplatin/paclitaxel	Progression
5	11	0	0	9	LN metastases with progression on hormonal therapy	Progression
5	12	0	0	1	LN metastases with progression on hormonal therapy and docetaxel currently on abiraterone/prednisone	Responding
6	*13	40	0	166	bone metastases with progression on docetaxel and investigational therapies	Progression
6	14	0	3	6	bone metastases with progression on docetaxel, investigational therapies, and abiraterone	Progression
7	15	2	1	41	bone, LN, colon, and bladder metastases following hormonal and investigational therapy	Progression
7	16	0	0	2	bone, LN, colon, and bladder metastases following hormonal and investigational therapy, currently on docetaxel	Responding
8	*17	0	2	3	bones metastases previously treated with multiple lines of hormonal therapy	Progression
8	18	0	0	0	bones metastases previously treated with multiple lines of hormonal therapy, currently on docetaxel	Responding
9	19	0	0	0	bone metastases progressing on initial hormonal therapy	Progression
10	20	2	1	53	LN and liver metastases previously treated with docetaxel currently on abiraterone	Progression
11	*21	9	2	28	bone metastases previously treated with docetaxel, ketoconazole, and investigational therapy	Progression
12	*22	1	0	2	LN metastases previously treated investigational therapy and docetaxel, currently on enzalutamide	Progression
13	23	0	0	0	bone and LN metastases on leuprolide and bicalutamide	Progression
14	24	0	0	20	bone and lungs metastases previously treated with hormonal therapy	Progression
15	*25	0	0	10	bone, LN, bladder and lung metastases previously treated with investigational therapy and docetaxel	Progression
16	26	0	0	0	bone and LN metastases previously treated with investigational therapy and docetaxel	Progression
17	27	1	0	22	LN and bladder metastases previously treatd with ketoconazole, docetaxel, investigational therapy, currently on cabazitaxel	Progression

LN= lymph node; Total means all J591-labeled CTCs; *No anti-CD45 depletion performed

Any patient with a treatment regimen of chemotherapy and CYP17 had corticosteroids

See the methods section for anti-CD45 depletion technique

### Expression of cell surface markers for CTC-Endothelial interactions

**Figure 3:**
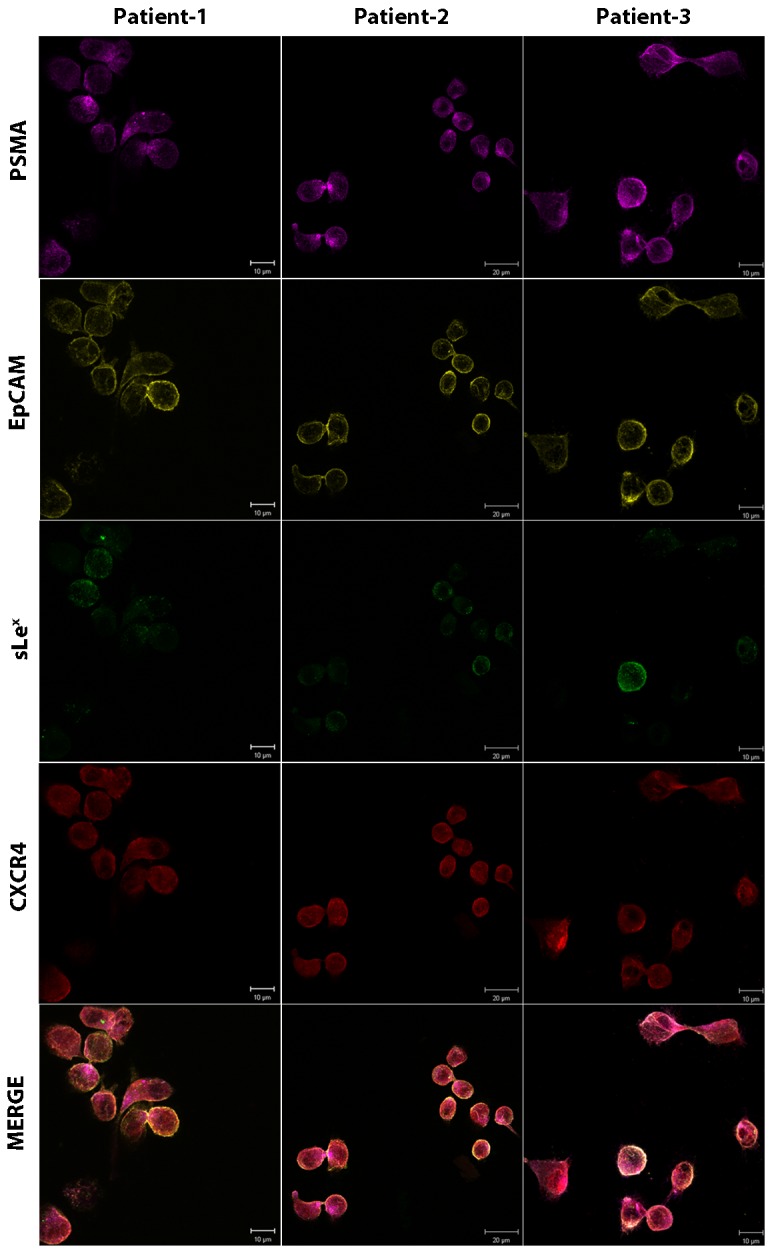
Isolation of prostate CTCs from metastatic PCa patients using anti-CD45 immunomagnetic depletion. 2.5 ml blood from three metastatic PCa patients (> 50 CTCs/ 2.5 ml blood) was processed via ficoll density centrifugation and the PBMC fraction was collected. Immunomagnetic anti-CD45 depletion was performed on the obtained PBMCs and the remaining cells were washed, cytospunned onto the slides. Slides were stained for PSMA, EpCAM, sLe^x^, and CXCR4 using the protocol as described in Figure S1. MDA, PC3, and KG1 cells were simultaneously stained as a control for the following markers: PSMA= Magenta, EpCAM= Yellow, HECA-452= Green, CXCR4= Red. All prostate CTCs expressed CXCR4, while, sLe^x^ expression was variable. The analysis of sLe^x^ intensity is shown in Figure 4.

**Figure 4:**
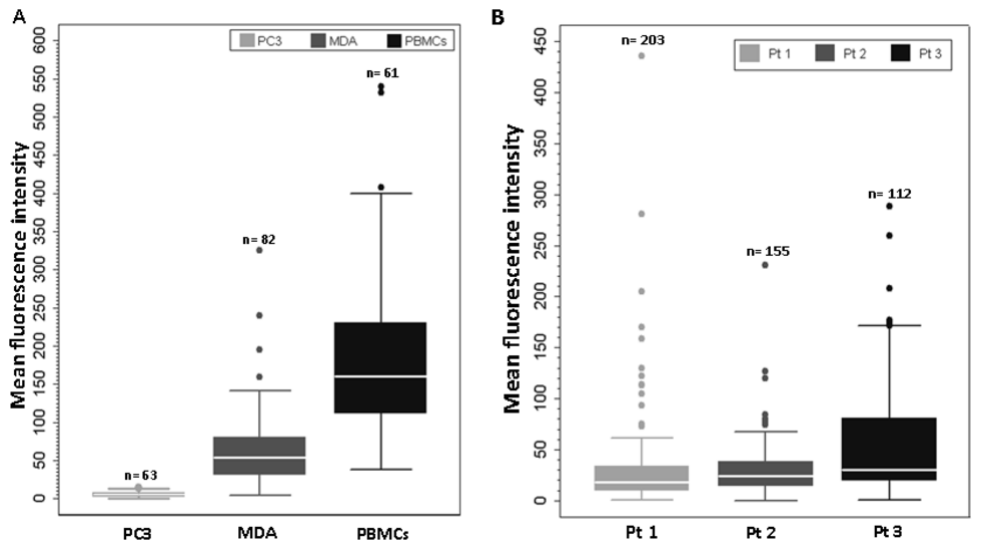
Box plot showing the percentage expression of sialyl Lewis X on human prostate CTCs. PBMCs from normal healthy donor blood, MDA, and PC3 cells were used as high expressors, moderate, and negative controls, respectively for determining sLe^x^ expression in metastatic PCa patients (sLe^x^ immunostaining shown in Fig. 3). **A**) MFI of sLe^x^ in PC3, MDA, and PBMCs. **B**) MFI of sLe^x^ in three CRPC patients with high CTC counts. Box plot shows that the median value for the MFI in PBMCs was 150 times higher than PC3 cells, and 100% of PBMCs expressed higher sLe^x^ intensity than PC3 cells. PC3 cells do not express sLe^x^ (range 0-20); therefore, MFI below 20 was considered negative for sLe^x^ expression. MDA cells have intermediate to high expression, 75% of the cells expressed sLe^x^ ranging from 50-140. Based on sLe^x^ expression in control cells, we determined the heterogenous expression of sLe^x^ in 3 prostate cancer patient CTCs with high CTC numbers. Comparing patient 3 and patient 1, 75% of the cells in patient 1, showed MFI ranging between 35-170; while in patient 1, it ranged from 15-60. The dots represent the outliers. n= number of cells counted for sLe^x^ expression. Pt= Patient number. Nil to low sLe^x^ expression = 0-20. Intermediate to Moderate expression = 20-100. High expression = >100.

E-selectin expressed on activated ECs interacts with a variety of ESL present on circulating leukocytes and tumor cells. These ESLs express a unique carbohydrate motif, sLe^x^ which appears to be required for ESL binding [28]. At the same time, chemokine receptor CXCR4 and its ligand CXCL12 mediate the adherence of prostate cancer cells to ECs, facilitating tumor invasiveness and metastatic progression. Xing et al. showed that stable downregulation of CXCR4 inhibits CXCL12-stimulated PCa adhesion to ECs [29]. In light of these studies, we decided to examine for the presence of both CXCR4 and sLe^x^ in prostate CTCs. After anti-CD45 depletion, PBMCs were immunostained for PSMA (to positively identify a prostate CTC), EpCAM (to confirm epithelial origin), CXCR4 and sLe^x^ (Figure 3). The conditions and imaging parameters for four color immunofluorescence were rigorously established using cell lines including MDA, PC3, KG1, as well as PBMCs obtained from healthy donor blood (Figures S1 and S2). These imaging parameters were then applied to patient derived CTCs. Immunostaining was performed on three of the patients with a relatively high CTC number as determined by CELLSEARCH^®^ CTC System (>50 CTCs/2.7 ml blood). Interestingly, 100% of the total CTCs (470/470) in these three patients expressed CXCR4; while KG1 cells (negative control) did not show any surface CXCR4 expression (Figure S1). In contrast, sLe^x^ antigen showed a wide range of expression levels in CTCs quantified by sLe^x^ MFI and compared to either leucocytes (highly positive), MDA cells (moderately positive), and PC3 cells (negative). As illustrated in Figure 4, 89/203 (43.8%; patient 1), 95/155 (61.3%; patient 2), and 84/112 (75%; patient 3) cells were moderately positive for sLe^x^ (MFI 20-100) while 5.4%, 1.9%, and 18.8%, cells, respectively, demonstrated high sLe^x^ expression (MFI >100). 

### Cytokine-mediated upregulation of E-selectin in HUVECs

**Figure 5:**
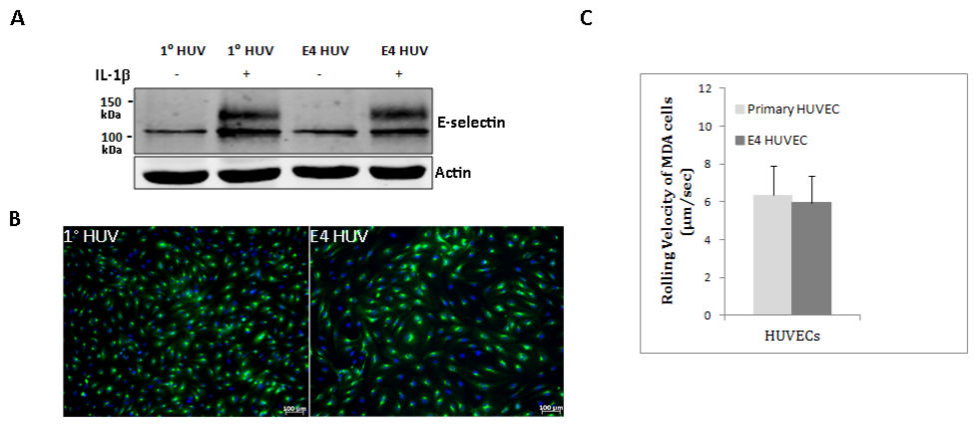
E-selectin expression and its functional assay in primary and E4ORF1 HUVECs. **A**) Western blot showing E-selectin protein expression in primary (1°) and E4ORF1 HUVECs. HUVECs were either stimulated with 50 ng/ml IL-1β for 4 h or left untreated. E-selectin= 95-115 kDa, based on post-translational modifications. The blot underneath E-selectin shows actin, used as a loading control. Western blot is a representative of three independent experiments. **B**) Immunofluorescence showing E-selectin expression in IL-1β-stimulated 1° HUVECs (passage=2) and E4ORF1 HUVECs (passage 10). E-selectin= Green, DAPI= Blue. **C**) Rolling velocity of 10^6^ MDA cells on 50 ng/ml IL-1β-stimulated 1°- and E4ORF1- HUVECs. HUVECs were stimulated with IL-1β for 4 h. The mean rolling velocity of MDA cells at 1 dyn/cm^2^ was 5.94 + 3.43 µm/sec and 6.35 + 3.92 µm/sec on E4-ORF1 and primary HUVECs, respectively. No significant difference was seen in the rolling velocities of MDA cells on either 1° or E4ORF1- HUVECs. Graph depicts Mean + SD.

To validate that E-selectin-mediated interactions occur physiologically, we used HUVECs as a biological model to demonstrate E-selectin-dependent adhesive interactions in tumor cells [30]. Upon cytokine stimulation with IL-1β or TNF-α, *de novo* protein synthesis of E-selectin occurs in HUVECs; whereas, unstimulated ECs express low levels of E-selectin [31]. Since conventional primary HUVECs have substantial technical constraints (e.g., early senescence in culture), we investigated the use of ECs expressing the adenoviral E4ORF1 gene to facilitate the study of CTC-E-selectin interactions [18]. Of note, E4ORF1-expressing HUVECs were previously shown to exhibit enhanced survival without causing cellular transformation, while the angiogenic function and growth factors-responsiveness were preserved [18]. We first compared the upregulation of E-selectin expression in primary and E4ORF1 HUVECs. Immunofluorescence and Western blotting both confirmed expression of E-selectin by HUVECs following a 4 h incubation with 50 ng/ml IL-1β (Figure 5 A and B). By western blotting, in the presence of IL-1β, two protein bands were detected between 100-130 KDa in both the HUVECs, with the higher molecular weight depicts the posttranslational modification, especially glycosylation [32]. In contrast, in unstimulated primary and E4ORF1 HUVECs, a minimal amount of E-selectin protein was detected by western blotting, whereas none was detectable by immunofluorescence (data not shown). We next performed a functional assay measuring the rolling behavior of MDA cells on both primary and E4ORF1 HUVECs using a parallel-plate flow chamber. Laminar flow chamber devices such as parallel-plate flow chamber are ideal to study cell-cell adhesive interactions [26]. The mean rolling velocity of MDA cells at 1 dyn/cm^2^ shear stress was 6.35 + 3.43 (primary HUVECS) and 5.94 + 3.92 µm/sec (E4ORF1 HUVECs), *p*=0.74, NS (Figure 5C). Therefore, E4ORF1 HUVECs (referred to hereafter simply as HUVECs) were used for further analyses based on their comparable E-selectin expression, similar capacity to support rolling, and convenience.

### E-selectin mediated-interactions between prostate cancer cells and ECs

**Figure 6:**
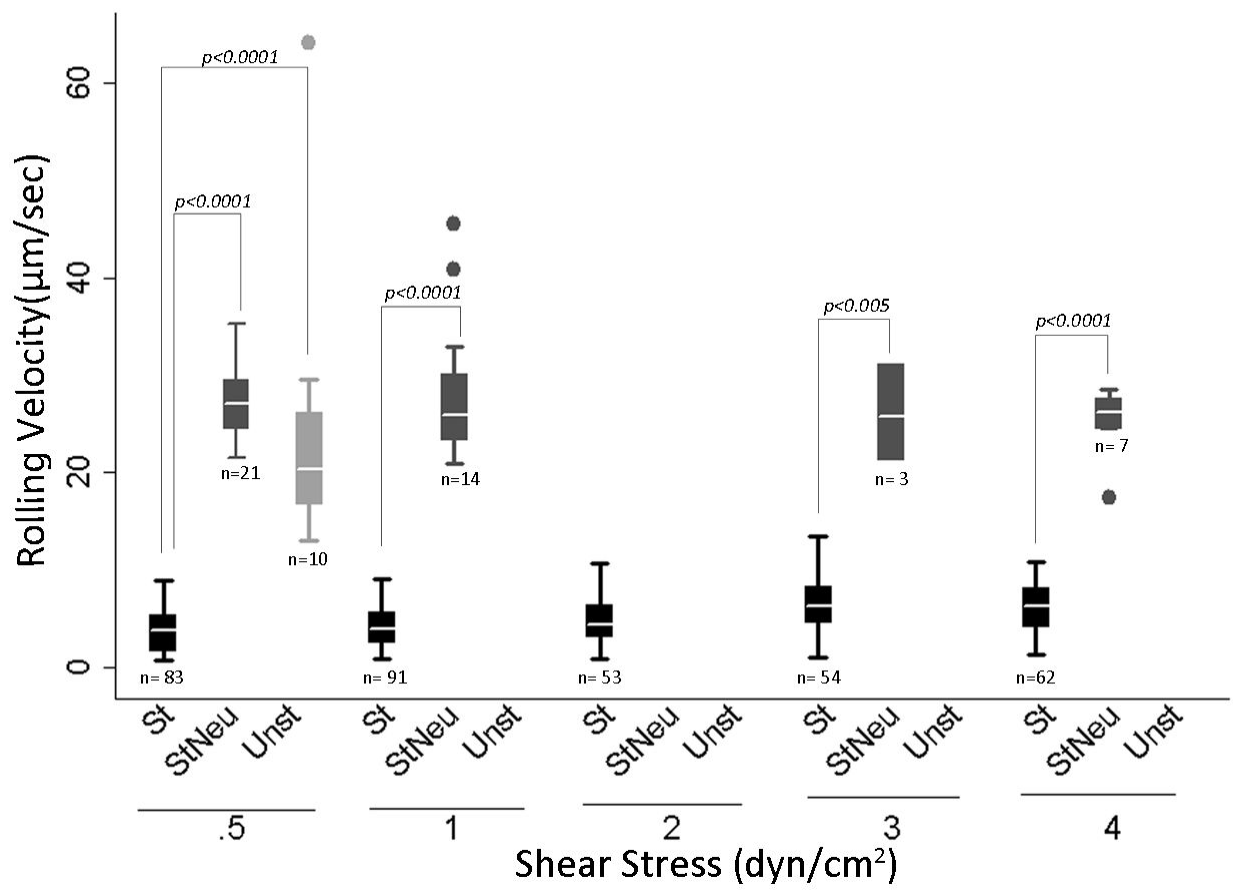
Box plot showing the rolling velocity of MDA cells on IL-1β-stimulated HUVECs. 1X10^6^ MDA cells were perfused over HUVECs. HUVECs used in the experiment were: *a*) stimulated with 50 ng/ml IL-1β for 4 h, *b*) unstimulated, and *c*) stimulated with 50 ng/ml IL-1β for 4h plus 1 h anti-E-selectin neutralizing antibody. Box plot shows the rolling velocities of MDA cells in three different categories of HUVECs. The rolling velocities in IL-1β-stimulated HUVECs ranged from 3.72+ 2.1 to 6.01 +2.45 µm/sec at different shear stresses. The dots represent the outliers. No box plot for the category means that no cells were found rolling at the mentioned shear stress. Box plots represent three separate experiments combined together. n= total number of cells rolling in 10 different fields at a given shear stress in three independent experiments. Notice the reduction in the number of cells (n) rolling in StNeu and Unst versus St category. St= IL-1β-stimulated HUVECs expressing E-selectin. StNeu= IL-1β-stimulated HUVECs incubated with anti-E-selectin neutralizing antibody. Unst= E4ORF1 HUVECs. *p*< 0.05 was considered significant.

To confirm that E-selectin mediates interactions between PCa cells and HUVECs, we measured the rolling behavior of MDA cells in the presence of neutralizing anti-E-selectin antibody. We performed these experiments using Bioflux Microfluidics technology by Fluxion Biosciences. Similar to the parallel-plate flow chamber, the Bioflux microfluidics system is a perfusion device providing a controlled laminar flow chamber to study cell-cell interactions [33]. The Bioflux system was selected at this point because of its reduced sample volume requirement and easier setup procedure compared with the parallel-plate flow chamber, allowing more flexibility while working with rare cells such as CTCs. MDA cells were perfused over HUVECs and the rolling velocity of MDA cells on HUVECs was determined under 3 conditions: a) unstimulated HUVECs, b) IL-1β-stimulated HUVECs, and c) IL-1β-stimulated HUVECs plus anti-E-selectin neutralizing Ab. The mean rolling velocity of MDA cells at 0.5 dyn/cm^2^ was 3.72 + 2.1µm/sec on IL-1β-stimulated HUVECs and 27.1 + 3.64 µm/sec on IL-1β-stimulated HUVECs plus anti-E-selectin neutralizing Ab, *p<0.0005* (Figure 6). This increase in the rolling velocity in the presence of E-selectin blocking antibody is consistent with the interpretation that the number of E-selectin binding sites has been significantly decreased and are unavailable for binding. Prior studies show a direct correspondence between rolling velocity and the surface density of available selectin binding sites [34,35]. Notably, a significant difference was observed in the rolling behavior between IL-1β-stimulated HUVECs and IL-1β stimulated HUVECs plus E-selectin neutralizing Ab (*p<0.005*) at all shear stresses ranging from 0.5-4 dyn/cm^2^ (Figure 6). In addition, there was a drastic decrease in the number of rolling cells in both of these conditions (Figure 6). Cells on unstimulated HUVECs (control) did not exhibit any rolling behavior at shear stresses ranging from 1-4 dyn/cm^2^; while at 0.5 dyn/cm^2^ shear stress, only 10 MDA cells were found rolling with a significantly higher rolling velocity than IL-1β stimulated HUVECs (*p <0.0005*). To exclude Fc-mediated interactions, IL-1β-stimulated HUVECs were incubated with anti-ICAM-1 antibody and the mean rolling velocities were found to be similar to IL-1β-stimulated HUVECs (p=0.27, NS, Figure S3A). The binding of anti-ICAM-1 antibody to HUVECs was confirmed by immunofluorescence (Figure S3B and C). Overall, these results strongly suggest that the expression of E-selectin in IL-1β-stimulated HUVECs is the primary determinant of rolling behavior in PCa cells.

### E-selectin-mediated interactions of prostate cancer CTCs with ECs

We next validated the role of E-selectin in CTC-endothelial interactions by perfusing prostate CTCs derived from patients over IL-1β-stimulated HUVECs incubated with anti-E-selectin neutralizing antibody. For these experiments, we first tested the Bioflux Microfluidics system. In our hands, the Bioflux microfluidic system was suboptimal for study of CTC-Endothelial interactions, as it introduced cell aggregation in the chamber, likely because of the presence of contaminating RBCs in PBMC preparations, which disturbed the laminar flow and made it difficult to assess (data not shown). Therefore, we used the parallel-plate flow chamber for further studies on CTCs. After anti-CD45 depletion and anti-PSMA J591-488 labeling, PBMCs were split into two equal fractions. One-half of each patient sample was perfused over IL-1β-stimulated HUVECs and the other half was perfused over IL-1β-stimulated HUVECs incubated with anti-E-selectin neutralizing antibody, at 0.6 dyn/cm^2^ shear stress. CTCs were detectable in 3 of 4 metastatic PCa patients analyzed. CTC-HUVEC interactions were predominant in IL-1β-stimulated HUVECs (median 5; range, 4-10); while in IL-1β-stimulated HUVECs in the presence of anti-E-selectin neutralizing antibody showed fewer CTC-HUVEC interactions or none at all (median 1; range, 0-3) (Table 2). After perfusion over HUVECs, we examined the flow-through of the patient samples collected in a dish to ensure the presence of CTCs. We observed the presence of CTCs (J591-488 labeled) in the flow-through of both IL-1β-stimulated HUVECs and IL-1β-stimulated HUVECs plus neutralizing anti-E-selectin antibody. Collectively, these results provide evidence that CTCs from PCa patients are capable of interacting with ECs specifically via E-selectin. 

**Table 2:** CTC-Endothelial interactions using parallel-plate flow chamber assay.

**Patient No.**	**No. of CTCs on E-sel HUV**	**No. of CTCs on E-sel HUV plus Neu Ab**
1	5	0
2	4	1
3	0	0
4	10	3

Number of CTCs on E-selectin expressing HUVECs; Number of CTCs on E-selectin expressing HUVECs plus E-selectin neutralizing antibody.

## Discussion

Studies examining the role of E-selectin in tumor metastasis typically use cultured human tumor cells [36,37] and suggest that E-selectin is critical to the metastatic process[7,9]. Patient derived tumor cells circulating in peripheral blood provide a unique reagent to study and validate this hypothesis. In this report, we demonstrate for the first time that CTCs derived from PCa patients’ peripheral blood can be studied *ex vivo*, and found physical interactions between CTCs and E-selectin under physiological shear flow. These results support the concept that prostate CTCs represent a dynamic tumor-derived population that allows real time tracking of their interactions with E-selectin expressing ECs.

A major limitation in studying human CTC-endothelial physical interactions has been the challenge of identifying and detecting low number of CTCs among millions of circulating blood cells. PSMA is expressed on virtually all prostate cancers, with the highest levels in metastatic lesions [38,39], thereby, presenting an attractive cell surface antigen to identify and label prostate CTCs. Anti-PSMA J591 antibody allowed the identification of prostate CTCs due to rapid internalization of anti-PSMA J591-488 antibody complex into the targeted cell following Ag-Ab binding [23]. Of note, others, including us, have used anti-PSMA J591 antibody to identify and characterize CTCs from human subjects [40,41]. 

By using MDA PCa cells, a cell line shown to interact with E-selectin, [17], we determined the effect of mAb J591-488 on rolling behavior. Under physiological flow conditions, both unlabeled and J591-488-labeled MDA cells showed comparable rolling, tethering and stable adhesion on both E-selectin-coated surface and E-selectin-expressing HUVECs. We then found that CTCs from PCa patients similarly demonstrated rolling/tethering and stable adhesions on E-selectin-coated surfaces. Selectins primarily promote transient adhesive interactions of tethering and rolling, and integrins tend to mediate firm adhesion, however, surprisingly CTC/E-selectin interactions were mainly of tethering and firm adhesive behavior rather than rolling adhesions under shear flow. This was observed both in patient samples perfused on E-selectin coated microtubes and on stimulated HUVECs. However, the CTC/E-selectin interactions observed over HUVECs included very small numbers of patient samples. In line with our observation, it has been reported that besides rolling behavior, different tumor types can firmly adhere on E-selectin independent of integrins [42-44]. Furthermore, Kitayama et al suggested that the firm arrest of tumor cells on E-selectin surfaces might be attributed to the distribution of selectin ligands on the cell surface [44]. This suggests that E-selectin can mediate both initial docking of CTCs, observed as rolling and tethering, and a further locking step, as observed by firm adhesion. Several studies provide evidence that the interaction between tumor cells and ECs expressing E-selectin is clinically significant because of its association with metastasis. Colon cancer cell clones showed a direct correlation between ability to bind to E-selectin expressing ECs and metastatic potential [4], [45], while a subpopulation of colon cancer cells initiated the process of diapedesis after adhering to activated HUVECs expressing E-selectin [46]. Given the importance of E-selectin-mediated interactions in tumor cells, where tumor cell/E-selectin interactions culminate in tumor cell transmigration, it can be postulated that PCa CTC/E-selectin adhesion may be the trigger required for the initiation of CTC transmigration and formation of a secondary metastatic niche. Notably, in our study, not all CTCs from the same patient exhibited interactions with E-selectin, suggesting a heterogeneous population of prostate CTCs having different phenotypic and functional characteristics. 

In the present report, it was also found that the clinical response of CRPC patients significantly correlates with CTC/E-selectin interactions, with no samples demonstrating interactions during period of clinical response, but many instances of interactions during periods of tumor progression. Interestingly, 4 paired samples that were collected on the same patients at times of tumor progression vs response demonstrated CTC/E-selectin interactions during progression that disappeared with successful systemic therapy resulting in clinical response. However, no conclusion can be drawn regarding E-selectin-mediated interactions and metastasis based upon these data alone. Prospective studies with a larger cohort of patients are required. Nevertheless, these results are consistent with our concept that CTC/E-selectin-mediated interactions can parallel the clinical response in CRPC patients.

The tethering and adhesion of CTCs with E-selectin suggests that prostate CTCs possess functional ESL(s) on their cell surface. E-selectin ligand activity on PCa cells is attributed to the expression of both sLe^x^ bearing glycoprotein and glycosphingolipids. Sialyl Lewis X is a terminal component of glycans attached to many proteins and lipids, and detected by HECA-452 antibody. Several studies showed that the presence of sLe^x^ epitope correlates with E-selectin ligand activity [47,48], however, it was subsequently established that sLe^x^ is necessary but not sufficient for ESL activity [10]. Based on the sLe^x^-reactive glycoproteins, various E-selectin ligand candidates have been identified. These include, but not all, the HECA-452 antigen bearing PSGL-1 (CLA), a glycoform of CD44 (HCELL), ESL-1, CD24, death receptor 3, L-selectin, and β2 integrins [1,28]. PSGL-1/CLA has been shown to bind all the three selectins and have been observed as the major E-selectin glycoprotein ligand present on prostate tumor cells [49]. Detection of sLe^x^ has shown a direct association between its presence and grade of prostate cancers [50,51]. Poorly differentiated prostate tumor cells exhibit a stronger sLe^x^ staining than normal prostate epithelial cells [17]. Based on immunocytochemical staining of an enriched population of CTCs, we found that a small percentage of CTCs in different patients expressed a considerably higher amount of sLe^x^, equivalent to sLe^x^ staining of PBMCs obtained from healthy donor blood. Similarly, immunohistochemical analysis of prostate tissue microarrays showed heterogeneous sLe^x^ staining with 38% of high-grade prostate tumors exhibiting high staining intensity, and only 15% of low-grade prostate tumors showing high sLe^x^ immunostaining [52]. Given the importance of sLe^x^ antigen to ESL activity, CTCs expressing high sLe^x^ may represent an aggressive subpopulation of CTCs that can potentially extravasate and metastasize to bone and other organs. Besides sLe^x^, there is a compelling evidence that chemokine receptors, such as CXCR4, also mediate metastasis [53]. In contrast to the heterogeneous expression of sLe^x^, we found that 100% of CTCs expressed CXCR4. Of note, a recent study evaluating the expression of CXCR4 on CTCs obtained from solid tumor patients showed that 82% of the patients expressed CXCR4; however, no correlation was found between the presence of CXCR4 and liver and lung metastasis [54]. 

In conclusion, our study confirms the presence of E-selectin-mediated interactions in PCa CTCs under physiologic shear stress conditions, providing a route to initiate the process of transendothelial migration and metastasis. Moreover, the significant correlation between the clinical response and CTC/E-selectin interactions are supportive of our pre-clinical hypothesis and justify additional prospective investigation. Although our study did not identify the precise ESL(s) present on CTCs that are necessary for E-selectin interactions, both the presence of sLe^x^ antigens and physical interaction of CTCs with E-selectin strongly indicate the presence of ESLs on prostate CTCs. Additional studies elucidating the expression of ESL(s) on PCa CTCs interacting with E-selectin compared with non-interacting PCa CTCs may help broaden our understanding of the metastatic heterogeneity and potentially provide a target to block metastatic spread. 

## Supporting Information

Figure S1 **Optimization of immunofluorescence staining of PSMA, EpCAM, HECA-452, and CXCR4 proteins.** MDA, PC3, KG1 cells were used for the optimization experiments. Cells were seeded onto cell-tak coated 48 mm coverslips in a 48-well plate. Cells were fixed with 2% formaldehyde for 20 min, washed with PBS. After fixation, cells were blocked with 2.0% BSA in PBS for 1 h at room temperature. All the cells were incubated with a primary antibody for anti-rabbit CXCR4 and anti-rat sLe^x^ for 1 h. After washing with PBS, cells were put in respective secondary antibodies-anti-rabbit dylight 405 and anti-rat AF594 for 1 h. Cells were then incubated with conjugated primary antibodies- humanized PSMA- AF488 and anti-mouse EpCAM- AF647 for 1 h. Cells were washed and mounted on a glass slide. PSMA= Magenta, EpCAM= Yellow, sLe^x^= Green, CXCR4= Red, and Merge shows all the colors.
MDA=PSMA+, EpCAM+, sLe^x^+, CXCR4+.
PC3 = PSMA-, EpCAM+, sLe^x^-, CXCR4+.
KG1 = PSMA-, EpCAM-, sLe^x^+, CXCR4-.
(TIF)

Figure S2 **Immunofluorescence staining of PBMCs obtained from normal healthy blood mixed with MDA, and PC3 cells.** PBMCs isolated from normal healthy donors were mixed with MDA and PC3 cells. Spiking experiments were conducted to observe the specificity of CTC markers (PSMA and EpCAM). After spiking, cells were seeded onto coverslips and stained as described in the methods and Figure S1. PSMA= Magenta, EpCAM= Yellow, sLe^x^= Green, CXCR4= Red, and Merge shows all the colors. The white arrowheads indicate PBMCs. Note that PBMCs lack the expression of PSMA and EpCAM, while PBMCs do express CXCR4 and sLe^x^.
(TIF)

Figure S3 **Effect of anti-ICAM1 antibody on the interactions between MDA cells and HUVECs.** **A**) Rolling velocity of MDA cells on IL-1β-stimulated HUVECs plus anti-ICAM-1 antibody. The mean rolling velocity of MDA cells between IL-1β-stimulated HUVECs plus anti-ICAM-1 antibody and IL-1β-stimulated HUVECs were measured. No significant difference was observed between the two groups (p = 0.27, Wilcoxon rank-sum test). **B** and **C**) Immunostaining of IL-1β-stimulated HUVECs plus anti-ICAM-1 antibody. HUVECs were stimulated with IL-1β for 4 h and human anti-ICAM-1 antibody was added @ 10 µg/ml for 1 h. MDA cells were perfused over the HUVECs and at the end of the perfusion, cells were washed and incubated with donkey anti-mouse Alexa fluor 488 secondary antibody. Cells were fixed and counterstained with DAPI. ICAM-1 (Green) and DAPI (Blue) analyzed by point scanning confocal microscopy. Scale Bar = 50 µm. (B) taken at 10X, while (C) taken at 20X.
(TIF)

Video S1 **A video showing rolling behavior in MDA cells through E-selectin coated microtubes.** 10^6^ MDA cells were perfused over microtube surfaces at 1 dyn/cm^2^ shear stress. The microtube was coated similarly as described in Materials and Methods. Ten videos were taken at different positions in the microtube. Robust rolling behavior in MDA cells is observed. The video is a representation of the rolling behavior in unlabeled MDA cells.
(AVI)

Video S2 **A video showing rolling behavior of J591-488 anti-PSMA antibody labeled-MDA cells on E-selectin coated microtubes.** 10^6^ MDA cells were perfused over E-selectin coated microtube surface at 1 dyn/cm^2^ shear stress. The microtube was coated similarly as described in Materials and Methods. Ten videos were taken at different positions in the microtube in fluorescence mode with 488 nm laser wavelength on an epifluorescence microscope (Zeiss Axiovert) using Zeiss Axiocam Mrm camera. The video is a representation of the rolling behavior in J591-488 labeled MDA cells.
(AVI)

Video S3 **A time stitched video showing tethering and stable adhesion in CTCs derived from one of the PCa patient.** A patient sample was perfused over the E-selectin coated surface and consecutive 45 sec videos were acquired for the total sample. This stitched video is a compilation of different videos of the same patient and same sample at different time points.
(AVI)

## References

[1] Läubli H, Borsig L (2010) Selectins promote tumor metastasis. Semin Cancer Biol 20: 169-177. doi:10.1016/j.semcancer.2010.04.005. PubMed: 20452433. 20452433

[2] Lawrence MB, Springer TA (1991) Leukocytes roll on a selectin at physiologic flow rates: Distinction from and prerequisite for adhesion through integrins. Cell 65: 859-873. doi:10.1016/0092-8674(91)90393-D. PubMed: 1710173. 1710173

[3] Petri B, Phillipson M, Kubes P (2008) The physiology of leukocyte recruitment: An in vivo perspective. J Immunol 180: 6439-6446. PubMed: 18453558. 18453558 10.4049/jimmunol.180.10.6439

[4] Laferriere J, Houle F, Taher MM, Valerie K, Huot J (2001) Transendothelial migration of colon carcinoma cells requires expression of E-selectin by endothelial cells and activation of stress-activated protein kinase-2 (SAPK2/p38) in the tumor cells. J Biol Chem 276: 33762-33772. doi:10.1074/jbc.M008564200. PubMed: 11448946. 11448946

[5] Khatib AM, Fallavollita L, Wancewicz EV, Monia BP, Brodt P (2002) Inhibition of hepatic endothelial E-selectin expression by C-raf antisense oligonucleotides blocks colorectal carcinoma liver metastasis. Cancer Res 62: 5393-5398. PubMed: 12359742. 12359742

[6] Khatib AM, Kontogiannea M, Fallavollita L, Jamison B, Meterissian S et al. (1999) Rapid induction of cytokine and E-selectin expression in the liver in response to metastatic tumor cells. Cancer Res 59: 1356-1361. PubMed: 10096570. 10096570

[7] Biancone L, Araki M, Araki K, Vassalli P, Stamenkovic I (1996) Redirection of tumor metastasis by expression of E-selectin in vivo. J Exp Med 183: 581-587. doi:10.1084/jem.183.2.581. PubMed: 8627169. 8627169 PMC2192458

[8] Kobayashi K, Matsumoto S, Morishima T, Kawabe T, Okamoto T (2000) Cimetidine inhibits cancer cell adhesion to endothelial cells and prevents metastasis by blocking E-selectin expression. Cancer Res 60: 3978-3984. PubMed: 10919677. 10919677

[9] Köhler S, Ullrich S, Richter U, Schumacher U (2010) E-/P-selectins and colon carcinoma metastasis: First in vivo evidence for their crucial role in a clinically relevant model of spontaneous metastasis formation in the lung. Br J Cancer 102: 602-609. doi:10.1038/sj.bjc.6605492. PubMed: 20010946. 20010946 PMC2822933

[10] Dimitroff CJ, Lee JY, Rafii S, Fuhlbrigge RC, Sackstein R (2001) CD44 is a major E-selectin ligand on human hematopoietic progenitor cells. J Cell Biol 153: 1277-1286. doi:10.1083/jcb.153.6.1277. PubMed: 11402070. 11402070 PMC2192031

[11] Sackstein R, Dimitroff CJ (2000) A hematopoietic cell L-selectin ligand that is distinct from PSGL-1 and displays N-glycan-dependent binding activity. Blood 96: 2765-2774. PubMed: 11023510. 11023510

[12] Idikio HA (1997) Sialyl-lewis-X, gleason grade and stage in non-metastatic human prostate cancer. Glycoconj J 14: 875-877. doi:10.1023/A:1018502424487. PubMed: 9511995. 9511995

[13] Kannagi R (2004) Molecular mechanism for cancer-associated induction of sialyl lewis X and sialyl lewis A expression-the warburg effect revisited. Glycoconj J 20: 353-364. PubMed: 15229399. 15229399 10.1023/B:GLYC.0000033631.35357.41

[14] Barthel SR, Hays DL, Yazawa EM, Opperman M, Walley KC et al. (2013) Definition of molecular determinants of prostate cancer cell bone extravasation. Cancer Res 73: 942-952. doi:10.1158/0008-5472.CAN-12-3264. PubMed: 23149920. 23149920 PMC3548951

[15] Liu H, Moy P, Kim S, Xia Y, Rajasekaran A et al. (1997) Monoclonal antibodies to the extracellular domain of prostate-specific membrane antigen also react with tumor vascular endothelium. Cancer Res 57: 3629-3634. PubMed: 9288760. 9288760

[16] Hughes AD, Mattison J, Western LT, Powderly JD, Greene BT et al. (2012) Microtube device for selectin-mediated capture of viable circulating tumor cells from blood. Clin Chem 58: 846-853. doi:10.1373/clinchem.2011.176669. PubMed: 22344286. 22344286

[17] Dimitroff CJ, Lechpammer M, Long-Woodward D, Kutok JL (2004) Rolling of human bone-metastatic prostate tumor cells on human bone marrow endothelium under shear flow is mediated by E-selectin. Cancer Res 64: 5261-5269. doi:10.1158/0008-5472.CAN-04-0691. PubMed: 15289332. 15289332

[18] Seandel M, Butler JM, Kobayashi H, Hooper AT, White IA et al. (2008) Generation of a functional and durable vascular niche by the adenoviral E4ORF1 gene. Proc Natl Acad Sci U S A 105: 19288-19293. doi:10.1073/pnas.0805980105. PubMed: 19036927. 19036927 PMC2588414

[19] Lee D, Schultz JB, Knauf PA, King MR (2007) Mechanical shedding of L-selectin from the neutrophil surface during rolling on sialyl lewis x under flow. J Biol Chem 282: 4812-4820. PubMed: 17172469. 17172469 10.1074/jbc.M609994200

[20] del Pozo MA, Alderson NB, Grande-GarcÃa A, Balasubramanian N, Schwartz MA et al. (2005) Phospho-caveolin-1 mediates integrin-regulated membrane domain internalisation. Nat Cell Biol 7: 901-908. doi:10.1038/ncb1293. PubMed: 16113676. 16113676 PMC1351395

[21] Tagawa ST, Milowsky MI, Morris MJ, Vallabhajosula S, Christos PJ et al. (2013) Phase II study of lutetium-177 labeled anti-prostate-specific membrane antigen (PSMA) monoclonal antibody J591 for metastatic castration-resistant prostate cancer. Clin Cancer Res 19: 5182-5191. doi:10.1158/1078-0432.CCR-13-0231. PubMed: 23714732. 23714732 PMC3778101

[22] Mannweiler S, Amersdorfer P, Trajanoski S, Terrett JA, King D et al. (2009) Heterogeneity of prostate-specific membrane antigen (PSMA) expression in prostate carcinoma with distant metastasis. Pathol Oncol Res 15: 167-172. doi:10.1007/s12253-008-9104-2. PubMed: 18802790. 18802790

[23] Liu H, Rajasekaran AK, Moy P, Xia Y, Kim S et al. (1998) Constitutive and antibody-induced internalization of prostate-specific membrane antigen. Cancer Res 58: 4055-4060. PubMed: 9751609. 9751609

[24] Hsu JW, Yasmin-Karim S, King MR, Wojciechowski JC, Mickelsen D et al. (2011) Suppression of prostate cancer cell rolling and adhesion to endothelium by 1alpha,25-dihydroxyvitamin D3. Am J Pathol 178: 872-880. doi:10.1016/j.ajpath.2010.10.036. PubMed: 21281819. 21281819 PMC3069912

[25] Yin X, Rana K, Ponmudi V, King MR (2010) Knockdown of fucosyltransferase III disrupts the adhesion of circulating cancer cells to E-selectin without affecting hematopoietic cell adhesion. Carbohydr Res 345: 2334-2342. doi:10.1016/j.carres.2010.07.028. PubMed: 20833389. 20833389 PMC2995892

[26] Remuzzi A, Giavazzi R (1999) Adhesion of tumor cells under flow. In: Dejana E Corada M Adhesion Protein Protocols. New Jersey: Humana Press pp. 153-157. 10.1385/1-59259-258-9:153 10098133

[27] Kim MB, Sarelius IH (2004) Regulation of leukocyte recruitment by local wall shear rate and leukocyte delivery. Microcirculation 11: 55-67. doi:10.1080/10739680490266199. PubMed: 15280097. 15280097

[28] Dimitroff CJ, Descheny L, Trujillo N, Kim R, Nguyen V et al. (2005) Identification of leukocyte E-selectin ligands, P-selectin glycoprotein ligand-1 and E-selectin ligand-1, on human metastatic prostate tumor cells. Cancer Res 65: 5750-5760. doi:10.1158/0008-5472.CAN-04-4653. PubMed: 15994950. 15994950 PMC1472661

[29] Xing Y, Liu M, Du Y, Qu F, Li Y et al. (2008) Tumor cell-specific blockade of CXCR4/SDF-1 interactions in prostate cancer cells by hTERT promoter induced CXCR4 knockdown: A possible metastasis preventing and minimizing approach. Cancer Biol Ther 7: 1839-1848. doi:10.4161/cbt.7.11.6862. PubMed: 18836306. 18836306

[30] Konstantopoulos K, Thomas SN (2009) Cancer cells in transit: The vascular interactions of tumor cells. Annu Rev Biomed Eng 11: 177-202. doi:10.1146/annurev-bioeng-061008-124949. PubMed: 19413512. 19413512

[31] Rahman A, Kefer J, Bando M, Niles WD, Malik AB (1998) E-selectin expression in human endothelial cells by TNF-alpha-induced oxidant generation and NF-kappaB activation. Am J Physiol 275: L533-L544. PubMed: 9728048. 9728048 10.1152/ajplung.1998.275.3.L533

[32] Bevilacqua MP, Stengelin S, Gimbrone MA Jr, Seed B (1989) Endothelial leukocyte adhesion molecule 1: An inducible receptor for neutrophils related to complement regulatory proteins and lectins. Science 243: 1160-1165. doi:10.1126/science.2466335. PubMed: 2466335. 2466335

[33] Conant CG, Nevill JT, Zhou Z, Dong JF, Schwartz MA et al. (2011) Using well-plate microfluidic devices to conduct shear-based thrombosis assays. J Lab Autom 16: 148-152. doi:10.1016/j.jala.2010.10.005. PubMed: 21609696. 21609696

[34] Rana K, Liesveld JL, King MR (2009) Delivery of apoptotic signal to rolling cancer cells: A novel biomimetic technique using immobilized TRAIL and E-selectin. Biotechnol Bioeng 102: 1692-1702. doi:10.1002/bit.22204. PubMed: 19073014. 19073014

[35] Rana K, Reinhart-King CA, King MR (2012) Inducing apoptosis in rolling cancer cells: A combined therapy with aspirin and immobilized TRAIL and E-selectin. Mol Pharm 9: 2219-2227. PubMed: 22724630. 22724630 10.1021/mp300073j PMC3412427

[36] Burdick MM, Chu JT, Godar S, Sackstein R (2006) HCELL is the major E- and L-selectin ligand expressed on LS174T colon carcinoma cells. J Biol Chem 281: 13899-13905. doi:10.1074/jbc.M513617200. PubMed: 16565092. 16565092

[37] Tremblay PL, Auger FA, Huot J (2006) Regulation of transendothelial migration of colon cancer cells by E-selectin-mediated activation of p38 and ERK MAP kinases. Oncogene 25: 6563-6573. doi:10.1038/sj.onc.1209664. PubMed: 16715142. 16715142

[38] Akhtar NH, Pail O, Saran A, Tyrell L, Tagawa ST. (2012) Prostate-specific membrane antigen-based therapeutics. Adv Urol 2012: 973820 10.1155/2012/973820 PMC3145341 21811498

[39] Osborne JR, Akhtar NH, Vallabhajosula S, Anand A, Deh K et al. (2013) Prostate-specific membrane antigen-based imaging. Urol Oncol 31: 144-154. doi:10.1016/j.urolonc.2012.04.016. PubMed: 22658884. 22658884 PMC3461099

[40] Darshan MS, Loftus MS, Thadani-Mulero M, Levy BP, Escuin D et al. (2011) Taxane-induced blockade to nuclear accumulation of the androgen receptor predicts clinical responses in metastatic prostate cancer. Cancer Res 71: 6019-6029. doi:10.1158/0008-5472.CAN-11-1417. PubMed: 21799031. 21799031 PMC3354631

[41] Miyamoto DT, Lee RJ, Stott SL, Ting DT, Wittner BS et al. (2012) Androgen receptor signaling in circulating tumor cells as a marker of hormonally responsive prostate cancer. Cancer Discov 2: 995-1003. Available online at: doi:10.1158/2159-8290.CD -12-0222 PubMed: 23093251. 23093251 10.1158/2159-8290.CD-12-0222 PMC3508523

[42] Burdick MM, McCaffery JM, Kim YS, Bochner BS, Konstantopoulos K (2003) Colon carcinoma cell glycolipids, integrins, and other glycoproteins mediate adhesion to HUVECs under flow. Am J Physiol Cell Physiol 284: C977-C987. doi:10.1152/ajpcell.00423.2002. PubMed: 12477667. 12477667

[43] Giavazzi R, Foppolo M, Dossi R, Remuzzi A (1993) Rolling and adhesion of human tumor cells on vascular endothelium under physiological flow conditions. J Clin Invest 92: 3038-3044. doi:10.1172/JCI116928. PubMed: 7504697. 7504697 PMC288509

[44] Kitayama J, Tsuno N, Sunami E, Osada T, Muto T et al. (2000) E-selectin can mediate the arrest type of adhesion of colon cancer cells under physiological shear flow. Eur J Cancer 36: 121-127. doi:10.1016/S0959-8049(99)00228-2. PubMed: 10741305. 10741305

[45] Sawada R, Tsuboi S, Fukuda M (1994) Differential E-selectin-dependent adhesion efficiency in sublines of a human colon cancer exhibiting distinct metastatic potentials. J Biol Chem 269: 1425-1431. PubMed: 7507108. 7507108

[46] Tremblay PL, Huot J, Auger FA (2008) Mechanisms by which E-selectin regulates diapedesis of colon cancer cells under flow conditions. Cancer Res 68: 5167-5176. doi:10.1158/0008-5472.CAN-08-1229. PubMed: 18593916. 18593916

[47] Rossiter H, van Reijsen F, Mudde GC, Kalthoff F, Bruijnzeel-Koomen CA et al. (1994) Skin disease-related T cells bind to endothelial selectins: Expression of cutaneous lymphocyte antigen (CLA) predicts E-selectin but not P-selectin binding. Eur J Immunol 24: 205-210. doi:10.1002/eji.1830240132. PubMed: 7517361. 7517361

[48] Fuhlbrigge RC, Kieffer JD, Armerding D, Kupper TS (1997) Cutaneous lymphocyte antigen is a specialized form of PSGL-1 expressed on skin-homing T cells. Nature 389: 978-981. doi:10.1038/40166. PubMed: 9353122. 9353122

[49] Barthel SR, Gavino JD, Descheny L, Dimitroff CJ (2007) Targeting selectins and selectin ligands in inflammation and cancer. Expert Opin Ther Targets 11: 1473-1491. doi:10.1517/14728222.11.11.1473. PubMed: 18028011. 18028011 PMC2559865

[50] Inaba Y, Ohyama C, Kato T, Satoh M, Saito H et al. (2003) Gene transfer of alpha1,3-fucosyltransferase increases tumor growth of the PC-3 human prostate cancer cell line through enhanced adhesion to prostatic stromal cells. Int J Cancer 107: 949-957. doi:10.1002/ijc.11513. PubMed: 14601054. 14601054

[51] Barthel SR, Wiese GK, Cho J, Opperman MJ, Hays DL et al. (2009) Alpha 1,3 fucosyltransferases are master regulators of prostate cancer cell trafficking. Proc Natl Acad Sci U S A 106: 19491-19496. doi:10.1073/pnas.0906074106. PubMed: 19889975. 19889975 PMC2780742

[52] Barthel SR, Gavino JD, Wiese GK, Jaynes JM, Siddiqui J et al. (2008) Analysis of glycosyltransferase expression in metastatic prostate cancer cells capable of rolling activity on microvascular endothelial (E)-selectin. Glycobiology 18: 806-817. doi:10.1093/glycob/cwn070. PubMed: 18647941. 18647941 PMC2574550

[53] Wang J, Loberg R, Taichman RS (2006) The pivotal role of CXCL12 (SDF-1)/CXCR4 axis in bone metastasis. Cancer Metastasis Rev 25: 573-587. PubMed: 17165132. 17165132 10.1007/s10555-006-9019-x

[54] Fusi A, Liu Z, Kümmerlen V, Nonnemacher A, Jeske J et al. (2012) Expression of chemokine receptors on circulating tumor cells in patients with solid tumors. J Transl Med 10: 52-5876-10-52 PubMed: 22433180. 22433180 10.1186/1479-5876-10-52 PMC3337808

