# Supplementary figures and images for: Circulating Tumor Cells from Prostate Cancer Patients Interact with E-Selectin under Physiologic Blood Flow
Source: PLoS One. 2013 Dec 27;8(12):e85143. doi: 10.1371/journal.pone.0085143 (PMC3874033; doi:10.1371/journal.pone.0085143)

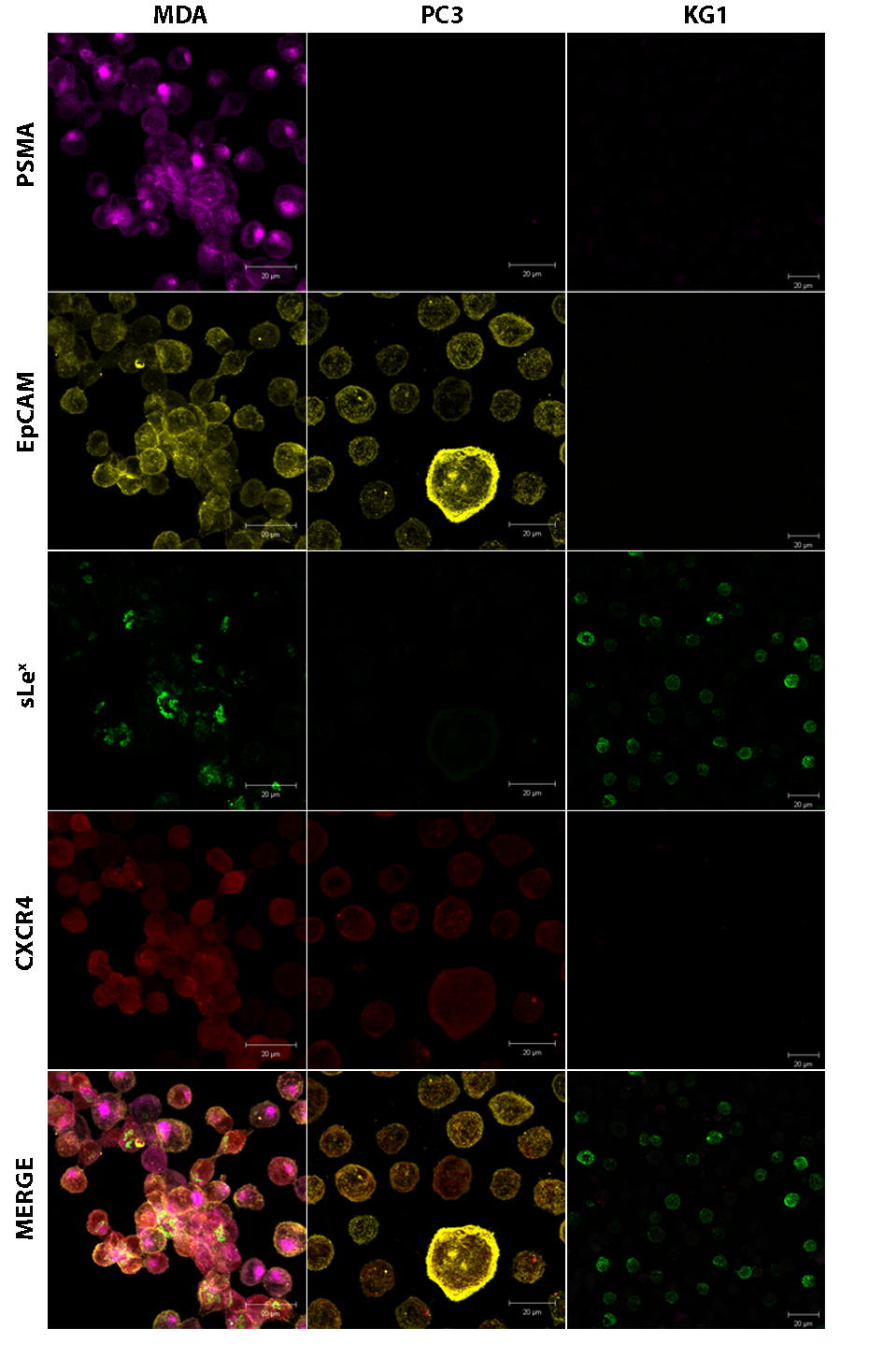

Supplement: Figure S1 — Optimization of immunofluorescence staining of PSMA, EpCAM, HECA-452, and CXCR4 proteins. MDA, PC3, KG1 cells were used for the optimization experiments. Cells were seeded onto cell-tak coated 48 mm coverslips in a 48-well plate. Cells were fixed with 2% formaldehyde for 20 min, washed with PBS. After fixation, cells were blocked with 2.0% BSA in PBS for 1 h at room temperature. All the cells were incubated with a primary antibody for anti-rabbit CXCR4 and anti-rat sLex for 1 h. After washing with PBS, cells were put in respective secondary antibodies-anti-rabbit dylight 405 and anti-rat AF594 for 1 h. Cells were then incubated with conjugated primary antibodies- humanized PSMA- AF488 and anti-mouse EpCAM- AF647 for 1 h. Cells were washed and mounted on a glass slide. PSMA= Magenta, EpCAM= Yellow, sLex= Green, CXCR4= Red, and Merge shows all the colors. MDA=PSMA+, EpCAM+, sLex+, CXCR4+. PC3 = PSMA-, EpCAM+, sLex-, CXCR4+. KG1 = PSMA-, EpCAM-, sLex+, CXCR4-. (TIF) [file pone.0085143.s001.tif]

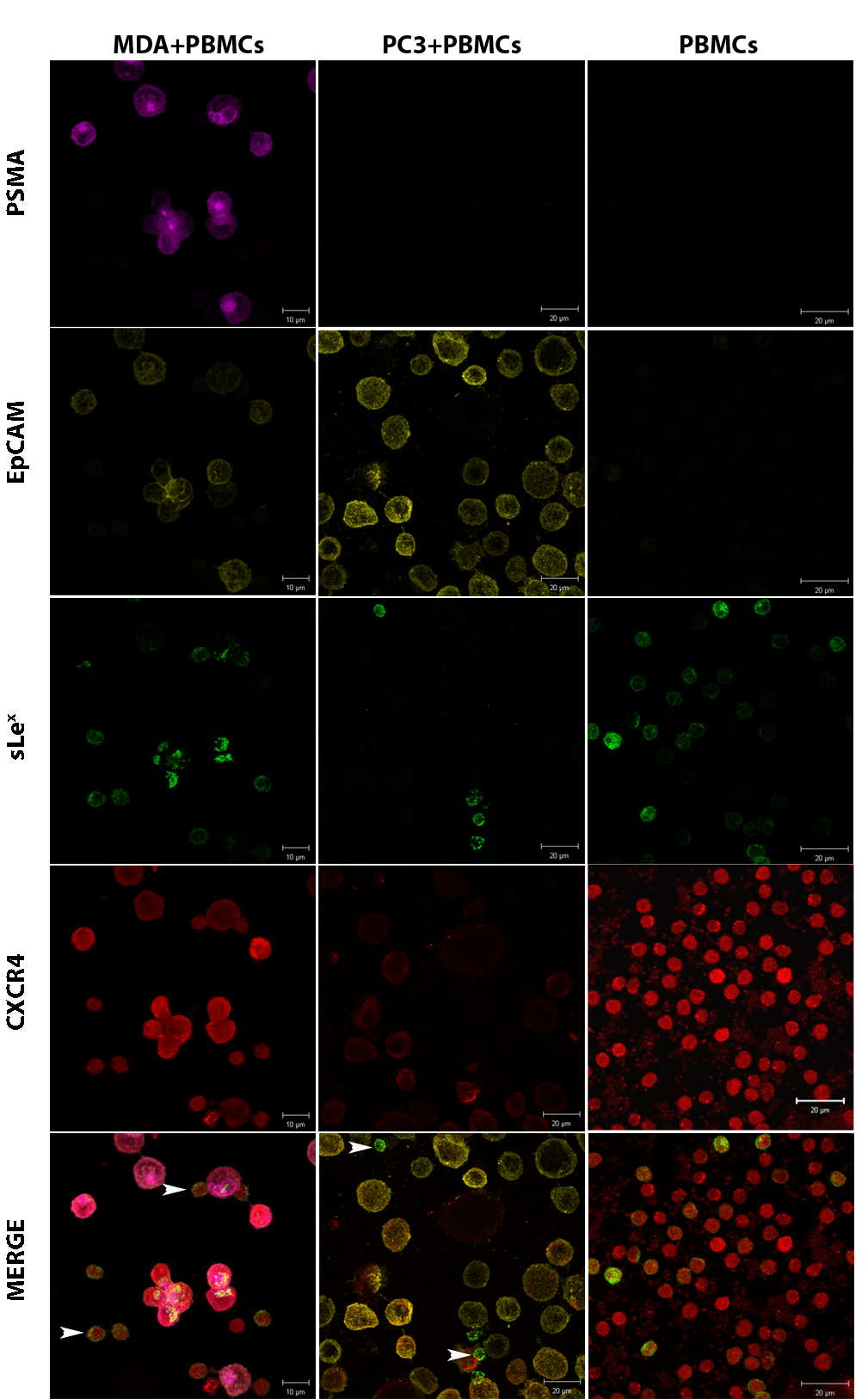

Supplement: Figure S2 — Immunofluorescence staining of PBMCs obtained from normal healthy blood mixed with MDA, and PC3 cells. PBMCs isolated from normal healthy donors were mixed with MDA and PC3 cells. Spiking experiments were conducted to observe the specificity of CTC markers (PSMA and EpCAM). After spiking, cells were seeded onto coverslips and stained as described in the methods and Figure S1. PSMA= Magenta, EpCAM= Yellow, sLex= Green, CXCR4= Red, and Merge shows all the colors. The white arrowheads indicate PBMCs. Note that PBMCs lack the expression of PSMA and EpCAM, while PBMCs do express CXCR4 and sLex. (TIF) [file pone.0085143.s002.tif]

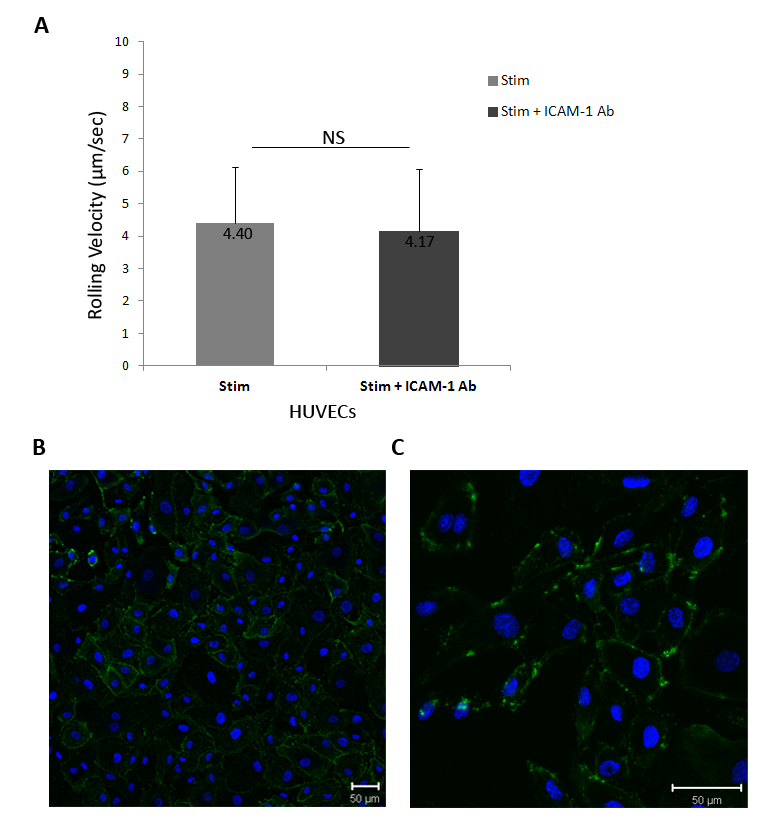

Supplement: Figure S3 — Effect of anti-ICAM1 antibody on the interactions between MDA cells and HUVECs. A) Rolling velocity of MDA cells on IL-1β-stimulated HUVECs plus anti-ICAM-1 antibody. The mean rolling velocity of MDA cells between IL-1β-stimulated HUVECs plus anti-ICAM-1 antibody and IL-1β-stimulated HUVECs were measured. No significant difference was observed between the two groups (p = 0.27, Wilcoxon rank-sum test). B and C) Immunostaining of IL-1β-stimulated HUVECs plus anti-ICAM-1 antibody. HUVECs were stimulated with IL-1β for 4 h and human anti-ICAM-1 antibody was added @ 10 µg/ml for 1 h. MDA cells were perfused over the HUVECs and at the end of the perfusion, cells were washed and incubated with donkey anti-mouse Alexa fluor 488 secondary antibody. Cells were fixed and counterstained with DAPI. ICAM-1 (Green) and DAPI (Blue) analyzed by point scanning confocal microscopy. Scale Bar = 50 µm. (B) taken at 10X, while (C) taken at 20X. (TIF) [file pone.0085143.s003.tif]
